# Molar Incisor Hypomineralization in Children: Prevalence and Risk Factors From a Cross-Sectional Study in Dong Thap, Vietnam

**DOI:** 10.7759/cureus.85104

**Published:** 2025-05-30

**Authors:** Tran Thi My Hanh, Pham Kim Thanh, Vo Truong Nhu Ngoc, Phan Thanh Tuong, Tran Tuan Anh, Lam Khanh Duy, Tran Duy Quan, Nguyen Thi Hai Anh

**Affiliations:** 1 School of Dentistry, Hanoi Medical University, Hanoi, VNM; 2 Faculty of Odonto-Stomatology, Can Tho University of Medicine and Pharmacy, Can Tho, VNM; 3 Department of Odonto-Stomatology, Can Tho Eye and Odonto-Stomatology Hospital, Can Tho, VNM; 4 Department of Odonto-Stomatology, Becamex International Hospital, Bình Dương, VNM; 5 Faculty of Odonto-Stomatology, University of Medicine and Pharmacy at Ho Chi Minh City, Ho Chi Minh City, VNM

**Keywords:** children, first molar, mih, molar incisor hypomineralization, permanent incisor, vietnam

## Abstract

Background

Molar incisor hypomineralization (MIH) is a common developmental enamel defect affecting the first permanent molars and incisors. It presents a range of clinical challenges, including hypersensitivity, enamel breakdown, and an increased risk of caries. In recent years, MIH has gained attention as a research topic, covering various aspects such as epidemiology, risk factors, impact on quality of life, and prevention and treatment guidelines. However, the epidemiology of MIH in Vietnam, particularly in the Southwest region, remains under-researched, with limited comprehensive studies.

Objective

This study aims to determine the prevalence of MIH and its associated risk factors in 7-10-year-old children in Dong Thap, Vietnam.

Methods

A cross-sectional study was conducted. Data were collected through clinical examination using the diagnostic criteria proposed by the European Academy of Paediatric Dentistry (EAPD) in 2010, along with a structured questionnaire. Statistical analysis was performed using Stata software (StataCorp LLC, USA, version 14.2).

Results

Among the 496 children examined, the prevalence of MIH was 22.2% (110 children). MIH status was not significantly associated with gender or age. Of the 110 children with MIH, 92.7% (102 children) had mild MIH, while 7.3% (8 children) had severe MIH. All affected children exhibited MIH in both the first molars and permanent incisors. A history of trauma to primary teeth was identified as a significant risk factor, increasing the likelihood of MIH by 1.73 times (adjusted OR: 1.73, 95% CI: 1.06-2.83).

Conclusion

The incidence of MIH among children in Dong Thap, Vietnam, is relatively high and is significantly associated with a history of trauma to primary teeth. Preventive measures and timely interventions are essential to minimize traumatic dental injuries in primary teeth and reduce the incidence of MIH.

Clinical significance

The prevalence of MIH in children aged 7-10 years in Dong Thap, Vietnam, is notably high. Among the examined risk factors, a history of traumatic dental injuries in primary teeth remained significantly associated with MIH, aligning with findings from previous studies. However, further long-term studies examining MIH risk factors from pregnancy through early childhood are needed in the future.

## Introduction

Molar incisor hypomineralization (MIH) is characterized as a hypomineralization of systemic origin. The characteristic symptom of MIH is the presence of opacities in the enamel of permanent incisors and molars [1]. One of the first studies on the occurrence of such distinct opacities in molars and incisors was reported by Koch G et al. in 1987 [2]. The first formal definition was provided by Weerheijm KL in 2001 [1], and the term MIH along with its definition is still in use today.

In recent years, MIH has become a widely studied topic, covering various aspects such as epidemiology, risk factors, impact on quality of life, prevention, and treatment guidelines [3-9]. According to a systematic review by Zhao D et al. (2018), the global prevalence of MIH ranges from 0.5% to 40.2%, with an average of 14.2% based on meta-analysis [10]. Similarly, Schwendicke F et al. (2019) reported a global average prevalence of 13.1%, which increases to 14.5% when considering only original studies using the European Academy of Paediatric Dentistry (EAPD) definition [11]. A 2021 study by Lopes LB et al. found an average MIH prevalence of 13.5%. The Americas reported the highest prevalence at 15.3%, while Asia had the lowest at 10.7% [4]. MIH studies in Southeast Asia are limited, with notable ones including the study by Pitiphat W et al. in Thailand in 2014, Ng JJ et al. in Singapore, and Hussein AS et al. in Malaysia in 2015, reporting MIH rates of 20%, 12.5%, and 16.9%, respectively [12-14].

In Vietnam, there are currently few studies on MIH. Recent findings indicate that the prevalence of MIH in Vietnam ranges from 8.35% to 20.1% across various provinces and cities [15-19]. Specifically, the prevalence in the Mekong Delta region is 13.32% in Vinh Long and 8.35% in Tra Vinh [15,17]. Additionally, some risk factors associated with MIH were identified, such as a history of respiratory diseases and dental caries in the study from Vinh Long [17], and a history of trauma to primary teeth and early tooth loss, according to the research by Vo TN and Hoang BD (2021) conducted in three provinces of Vietnam: Binh Dinh, Thanh Hoa, and Hai Phong [16].

Dong Thap is a centrally located province within the Mekong Delta region (Southwest region) of Vietnam, with relatively developed socio-economic and cultural conditions. To our knowledge, no studies on MIH have yet been conducted in this province, making it a meaningful location for expanding scientific evidence in this geographical area. Its accessibility also facilitated effective coordination and data collection. Therefore, this study aims to investigate the current prevalence of MIH and its associated risk factors among children in Dong Thap, Vietnam.

## Materials and methods

Research subjects

Children residing in Dong Thap, Vietnam, from September 2023 to May 2024.

Inclusion criteria

Children aged 7-10 years who were studying at one of five primary schools in Dong Thap Province, located in the Southwest region of Vietnam, and were present on the day of the examination were included in the study. Eligible participants were required to have at least one permanent molar or incisor. Additionally, both the children and their parents were informed about the study and provided consent to participate.

Exclusion criteria

Children who did not cooperate during any part of the examination or whose parents were unable to recall their medical history or provide responses to the questionnaire were excluded from the study.

Sample size

The sample size was calculated using the formula for estimating a population proportion with absolute precision:

$n = Z^{2}_{1-\frac{\alpha}{2}}.\frac{p.(1-p)}{d^{2}}$

In this formula:

n is the minimum required sample size.

α is the alpha error or Type-I error or significance level or false positive error, which is the probability of falsely rejecting the null hypothesis when it is true. We chose as 0.05, which corresponds to a 95% CI (Z = 1.96).

p is the expected prevalence expressed in proportion and this value is taken from the previous published studies. We chose p = 0.2, which corresponds to the incidence of MIH in Thai children according to a study published in 2014 [12].

d is the absolute precision or margin of error, set at 0.05.

Applying the formula, the minimum required sample size was 246 children. However, the study included data from 496 children across five schools.

Research methods

A cross-sectional study was conducted.

Diagnostic criteria

The diagnostic criteria used in this study were based on the guidelines proposed by the EAPD in 2010 [8], which remain the standard reference for MIH research.

Mild MIH

Opaque plaques appear on molars or incisors without post-eruptive enamel breakdown. No tooth sensitivity or aesthetic complaints are reported.

Severe MIH

Post-eruptive enamel breakdown, destruction of the tooth body, caries involving the affected tooth, history of tooth sensitivity, and aesthetic complaints.

MIH was assessed on the occlusal, buccal, lingual, and lateral surfaces of permanent molars and incisors. Lesions were recorded only if they were larger than 1 mm, in accordance with EAPD guidelines [8]. A diagnosis of MIH was made if at least one permanent molar or incisor was affected by hypomineralization. This classification excluded cases of enamel defects due to trauma and other conditions with similar features (e.g., fluorosis, enamel hypoplasia, amelogenesis imperfecta, or early-stage caries).

Standardization training

A training session on basic oral examination techniques and MIH detection was conducted at Phuong Thanh Dental Center. The session included four members of the research team, four general dentists, and one pediatric dentist with extensive experience in diagnosing and managing MIH. The pediatric dentist first provided instruction on MIH diagnostic criteria based on the EAPD 2010 guidelines [8]. Subsequently, 30 sets of intraoral photographs were reviewed to standardize classification into categories (no MIH, mild MIH, severe MIH) and to aid in the differential diagnosis of similar conditions such as fluorosis, enamel hypoplasia, amelogenesis imperfecta, and early-stage caries. Finally, a pilot examination was conducted with 20 children aged 7-10 years to provide the team with hands-on experience in identifying and diagnosing MIH in a community setting.

Clinical examination process

To record and describe the condition of enamel hypomineralization in molars and incisors, all permanent molars and incisors were examined in sequence, starting from the upper right quadrant, followed by the upper left, lower left, and lower right quadrants, under natural light and a headlamp. Teeth were examined in their natural moist condition; however, when necessary, they were cleaned and dried using gauze and compressed air. Dental mirrors and periodontal probes were used for the examination.

Data collection

After completing the standardization training course, the dentists examined the enamel condition of the molars and incisors of the participating students. All findings were thoroughly recorded on the examination forms. Simultaneously, questionnaires were distributed to the students’ parents to collect data on birth weight (to classify as underweight or normal), gestational age, duration of exclusive breastfeeding (categorized as up to 6 months or up to 12 months), early childhood medical history, and dental trauma history.

Data processing and analysis

Data were entered and processed using Microsoft Excel for Office 365 (Microsoft Corporation, USA, version 15.13.1) and analyzed using Stata software (StataCorp LLC, USA, version 14.2). Mean values and standard deviations were used to present quantitative variables with normal distribution. Frequencies and percentages were used to describe qualitative variables. Statistical tests such as unpaired t-tests, chi-square tests, and logistic regression analysis were conducted, with significance set at p < 0.05.

Ethical consideration

The Ethics Council for Biomedical Research of Can Tho University of Medicine and Pharmacy reviewed and approved the research proposal under license number 23.319.HV/PCT-HDDD, signed on April 12, 2023. In addition, the study received implementation permission from the Local Board of Directors of Medical and Educational Management. All children and their parents were informed about the research and confirmed their voluntary participation by signing the consent form.

## Results

The study involved 496 children between the ages of 7 and 10 who attended primary schools in Dong Thap, Vietnam, with a mean age of 8.50 ± 1.11 years. Males had a slightly higher mean age than females (8.53 ± 1.08 vs. 8.47 ± 1.13), but this difference was not statistically significant (unpaired t-test, p = 0.517). The sample included 250 boys (50.4%) and 246 girls (49.6%), evenly distributed across four age groups: 7-year-olds, 120 (24.2%); 8-year-olds, 130 (26.2%); 9-year-olds, 124 (25.0%); and 10-year-olds, 125 (24.6%). No statistically significant differences in gender or age distribution were found in the sample (Chi-square test, p = 0.308).

Of the 496 children who participated, 110 had MIH, accounting for 22.2% of the total. The study recorded a total of 335 teeth affected by MIH. The MIH status is presented in Tables 1-3, while the various risk factors associated with the condition are detailed in Tables 4-5.

**Table 1:** Characteristics of research subjects (n = 496). Statistical analysis was performed using the chi-square test. MIH: Molar incisor hypomineralization.

Characteristics	MIH n (%)	Non-MIH n (%)	Total n (%)	P-value
Gender				0.904
Male	56 (50.9)	194 (50.3)	250 (50.4)	
Female	54 (49.1)	192 (49.7)	246 (49.6)	
Age				0.066
7 years old	16 (14.6)	104 (26.9)	120 (24.2)	
8 years old	33 (30.0)	97 (25.1)	130 (26.2)	
9 years old	31 (28.2)	93 (24.1)	124 (25.0)	
10 years old	30 (27.3)	92 (23.8)	122 (24.6)	
Total	110 (100.0)	386 (100.0)	496 (100.0)	

**Table 2:** Distribution of MIH lesion severity by gender, age, and tooth type (n = 110). MIH: Molar incisor hypomineralization.

Characteristic	Mild MIH n (%)	Severe MIH n (%)	Total n (%)
Gender			
Male	51 (91.1)	5 (8.9)	56 (100.0)
Female	51 (94.4)	3 (5.6)	54 (100.0)
Age			
7 years old	15 (93.7)	1 (6.3)	16 (100.0)
8 years old	33 (100.0)	0 (0.0)	33 (100.0)
9 years old	26 (83.9)	5 (16.1)	31 (100.0)
10 years old	28 (93.3)	2 (6.7)	30 (100.0)
Tooth type			
Incisor	102 (92.7)	8 (7.3)	110 (100.0)
Molar	103 (93.6)	7 (6.4)	110 (100.0)
Total	102 (92.7)	8 (7.3)	110 (100.0)

**Table 3:** Distribution of MIH-affected teeth by severity level and tooth type (n = 335). MIH: Molar incisor hypomineralization.

Characteristics	Level of MIH	
	Mild MIH n (%)	Severe MIH n (%)
Tooth type		
Molar	139 (93.9)	9 (6.1)
Incisor	170 (90.9)	17 (9.1)
Total	309 (92.2)	26 (7.8)

**Table 4:** Association of risk factors and Molar Incisor Hypomineralization (MIH) (n=496) Univariable Logistic Regression Analysis △: Reference category MIH: Molar incisor hypomineralization.

Characteristics	MIH n (%)	Non-MIH n (%)	Total n (%)	Crude OR (95% CI)	P-value
Newborn birth-weight					0.015
Normal△	79 (71.8)	318 (82.4)	397 (80.0)	1	
Underweight	31 (28.2)	68 (17.6)	99 (20.0)	1.84 (1.12-3.00)	
Gestation length					0.01
Normal△	85 (77.3)	337 (87.3)	422 (85.1)	1	
Small for Gestational Age (SGA)	25 (22.7)	49 (12.7)	74 (14.9)	2.02 (1.18-3.46)	
Breast-fed exclusively duration					0.386
Up to 12 months△	57 (51.8)	218 (56.5)	275 (55.4)	1	
The first 6 months	53 (48.2)	168 (43.5)	221 (44.6)	1.21 (0.79-1.84)	
Digestive diseases					0.076
No△	68 (61.8)	273 (70.7)	341 (68.8)	1	
Yes	42 (38.2)	113 (29.3)	155 (31.2)	1.49 (0.96-2.32)	
Respiratory diseases					0.662
No△	47 (42.7)	174 (45.1)	221 (44.6)	1	
Yes	63 (57.3)	212 (54.9)	275 (55.4)	1.10 (0.72-1.69)	
Traumatic dental injuries in primary teeth					0.011
No△	76 (69.1)	311 (80.6)	387 (78.0)	1	
Yes	34 (30.9)	75 (19.4)	109 (22.0)	1.86 (1.15-2.99)	
Total n (%)	110 (100.0)	386 (100.0)	496 (100.0)		

**Table 5:** Association of risk factors with molar incisor hypomineralization (MIH) based on multivariable logistic regression analysis (n = 496). Multivariable logistic regression. △: Reference category MIH: Molar incisor hypomineralization.

Characteristics		MIH n (%)	Non-MIH n (%)	Total n (%)	Adjusted OR (95% CI)	P-value
Newborn birth-weight						0.052
Normal^△^	79 (71.8)		318 (82.4)	397 (80.0)	1 1.67 (0.99-2.79)	
Underweight	31 (28.2)		68 (17.6)	99 (20.0)		
Gestation length						0.129
Normal^△^	85 (77.3)		337 (87.3)	422 (85.1)	1 1.56 (0.88-2.77)	
Small for Gestational Age (SGA)	25 (22.7)		49 (12.7)	74 (14.9)		
Traumatic dental injuries in primary teeth						0.029
No^△^	76 (69.1)		311 (80.6)	387 (78.0)	1 1.73 (1.06-2.83)	
Yes	34 (30.9)		75 (19.4)	109 (22.0)		
Total n (%)	110 (100.0)		386 (100.0)	496 (100.0)		

As shown in Table 1, 110 out of 496 children (22.2%) were found to have MIH. Among these 110 children, 56 were male (50.9%) and 54 were female (49.1%). They were predominantly in the 8-year-old group (33 children, 30.0%) and the 9-year-old group (31 children, 28.2%), followed by the 10-year-old group (30 children, 27.3%) and the 7-year-old group (16 children, 14.6%). However, there was no statistically significant association between MIH status and either gender or age (Chi-square test, p = 0.904 and p = 0.066, respectively).

According to the data in Table 2, MIH was predominantly observed at a mild level, affecting 102 children (92.7%). Only 8 children (7.3%) were diagnosed with severe MIH. Regarding tooth location, mild MIH was most commonly detected in both molars and incisors, with reported rates of 93.6% (103 molars) and 92.7% (102 incisors), respectively. By gender, the proportion of males presenting with severe MIH was 8.9% (5 children), which was higher compared to females at 5.6% (3 children). Additionally, the age group with the highest prevalence of severe MIH was 9 years old, with 5 affected children, representing 16.1% of the severe MIH cases.

Upon further clinical examination of the 110 children diagnosed with MIH, a total of 335 affected teeth were identified. The characteristics of teeth affected by MIH, in relation to severity and tooth type, are presented in Table 3. Of the total, 309 teeth (92.2%) were classified as having mild MIH, while only 26 teeth (7.8%) exhibited severe MIH. When considering the location of the affected teeth, the prevalence of severe MIH was higher in incisors (9.1%) compared to molars (6.1%).

Univariate logistic regression analysis was carried out to identify risk factors associated with MIH. As shown in Table 4, being Small for Gestational Age (SGA), having a low birth weight, and a history of primary tooth trauma were all associated with an increased risk of MIH in 7- to 10-year-old children in Dong Thap. Specifically, children with low birth weight had 1.84 times higher odds of having MIH (crude OR: 1.84; 95% CI: 1.12-3.00), and those born SGA had 2.02 times higher odds (crude OR: 2.02; 95% CI: 1.18-3.46), with p-values of 0.015 and 0.010, respectively. Additionally, children with traumatic dental injuries to primary teeth had 1.86 times higher odds of MIH compared to those without (crude OR: 1.86; 95% CI: 1.15-2.99), with a corresponding p-value of 0.011. No statistically significant associations were found between MIH and other risk factors such as exclusive breastfeeding duration or a history of systemic diseases (e.g., digestive or respiratory illnesses), with p-values greater than 0.05.

As presented in Table 5, among the three risk factors identified in the univariate analysis, only traumatic dental injuries to primary teeth remained statistically significant in the multivariate logistic regression model. Specifically, these injuries increased the risk of MIH by 1.73 times (adjusted OR: 1.73; 95% CI: 1.06-2.83), with an adjusted p-value of 0.029.

## Discussion

The study was conducted on 496 children aged 7-10 years in Dong Thap, Vietnam. The authors Garg N et al. (2012) suggested that this age group is suitable for studying MIH because most children have fully erupted first molars and permanent incisors and have not been in the oral environment long enough to develop dental caries [20]. In addition, first molars usually maintain their shape well with a low rate of trauma [20]. Our study recorded a prevalence of MIH of 22% (110 children) according to EAPD criteria (2010) [8]. This classification excludes MIH due to trauma and diseases with similar characteristics (fluorosis, enamel hypoplasia, enamel imperfecta, early-stage caries). Although this result is within the range of 0.5% to 40.2%, as reported in the systematic review by Zhao D et al. [10], it is higher than the global average prevalence of MIH (14.5%) found in the meta-analysis by Schwendicke F et al. [11]. When compared with countries in Asia in general and Southeast Asia in particular, our results are similar to those of Pitiphat [12] in Thailand, who reported an MIH prevalence of 20%, but higher than the studies by Ng [13] in Singapore and Hussein [14] in Malaysia, with MIH prevalence of 12.5% and 16.9%, respectively. On the other hand, our results are lower than those reported by Alhowaish L et al. [21] in Saudi Arabia and Elzein R et al. [22] in Lebanon, with MIH prevalence of 40.5% and 26.7%, respectively. In Vietnam, our results are similar to those of Vo TN and Hoang BD [16] in 2021 in Binh Dinh, Thanh Hoa, and Hai Phong, who reported an MIH prevalence of 20.1%, but significantly higher than studies conducted in some other provinces and cities. Specifically, surveys in Tra Vinh, Vinh Long, Lao Cai, and Hanoi showed that the prevalence of MIH ranged from 8.35% to 13.32% [15,17-19].

Among children identified with MIH, our study found no statistically significant difference in the incidence between males and females, with rates of 50.9% and 49.1%, respectively. This finding is consistent with results from numerous previous studies worldwide, including two systematic reviews and meta-analyses by Zhao D et al. (2018) [10] and Lopes LB et al. (2021) [4]. Similar outcomes have also been reported in studies conducted in Southeast Asia and Vietnam [12-14,16,18,19]. In terms of MIH severity, our study showed that the incidence of severe MIH was only 7.3%, which is notably lower than that reported in most other studies. We also found that MIH affected 100% of incisors, with every child diagnosed with MIH according to EAPD criteria having at least one first molar and one permanent incisor affected [8]. This rate is significantly higher than those reported in most studies globally, regionally, and within Vietnam.

In our study, univariate logistic regression analysis identified risk factors associated with MIH, including SGA, low birth weight, and traumatic dental injuries in primary teeth. Subsequently, multivariate logistic regression analysis confirmed that only traumatic dental injuries in primary teeth increased the risk of MIH in 7-10-year-old children in Dong Thap, with the risk increasing by 1.73 times. This result is partly similar to the findings of a study conducted in Binh Dinh, Thanh Hoa, and Hai Phong by Vo TN and Hoang BD in 2021 [16]. According to that study, a history of trauma to primary teeth increased the risk of MIH in children by 1.12 times, in addition to early loss of primary teeth, which had a corresponding odds ratio of 1.26. Traumatic dental injuries in primary teeth are also a known risk factor for enamel hypomineralization, highlighting the importance of prevention. Regarding risk factors, our results differ somewhat from those of other studies conducted both in Vietnam and globally, which have not found a statistically significant relationship between MIH and factors such as SGA, low birth weight [5], or a history of systemic diseases [17]. Therefore, our research team agreed that a study involving a larger sample size is needed to better detect risk factors in the Vietnamese population, from which appropriate preventive measures can be developed.

The relatively high proportion of MIH cases associated with traumatic dental injuries underscores an urgent need to strengthen school-based health programs. These should emphasize injury prevention, early detection, and timely management of dental trauma among children. As a proactive step, our team has already organized a workshop in collaboration with local health and education authorities to raise awareness and promote preventive measures in school settings.

Our study relied on questionnaires completed by parents regarding their children’s early life medical history, which may be subject to inaccuracy and potential information bias. In addition, the study assessed MIH at a single point in time and did not track its progression or evaluate long-term outcomes, limitations that would require longitudinal studies in the future. However, our research team is currently conducting multiple studies on MIH in different regions of Vietnam, focusing not only on epidemiology but also on the immediate and long-term treatment outcomes of MIH-affected teeth using minimally invasive approaches.

## Conclusions

The prevalence of MIH among children aged 7-10 years in Dong Thap, Vietnam, is notably high. A history of trauma to primary teeth has been identified as a significant risk factor for MIH in this age group. This highlights the importance of timely prevention and intervention strategies to reduce dental trauma in early childhood and, in turn, lower the prevalence of MIH. Furthermore, there is a need for more long-term studies investigating MIH risk factors from pregnancy through early childhood.

## Appendices

Appendix 1

Section 1: General Information

Child’s full name: __________________________________________

Child’s date of birth (DD/MM/YYYY): ____ / ____ / ________

Gender: ☐ Male ☐ Female

Section 2: Perinatal Information

What was your child’s birth weight? ☐ Less than 2.5 kg (underweight) ☐ 2.5 kg or more (normal) ☐ Don’t remember

Was your child small for gestational age (SGA)? ☐ Yes  ☐ No  ☐ Don’t know

How long was your child exclusively breast-fed? ☐ Up to 6 months ☐ Up to 12 months ☐ Not breast-fed ☐ Don’t remember

Section 3: Medical History

Did your child have any digestive diseases during the first 3 years of life (e.g. chronic diarrhea, poor absorption, stomach issues)? ☐ Yes  ☐ No  ☐ Not sure

Did your child have any respiratory diseases during the first 3 years (e.g. asthma, bronchitis, pneumonia)? ☐ Yes  ☐ No  ☐ Not sure

Section 4: Dental History

Has your child ever had trauma (accident or injury) to baby teeth (primary teeth)? ☐ Yes ☐ No ☐ Don’t know

If yes, please describe briefly (e.g., fall, chipped tooth):
